# Impact of Full Vaccination with mRNA BNT162b2 on SARS-CoV-2 Infection: Genomic and Subgenomic Viral RNAs Detection in Nasopharyngeal Swab and Saliva of Health Care Workers

**DOI:** 10.3390/microorganisms9081738

**Published:** 2021-08-14

**Authors:** Michela Deiana, Antonio Mori, Chiara Piubelli, Francesca Perandin, Davide Treggiari, Davide Martini, Fabio Chesini, Andrea Angheben, Francesco Bonfante, Calogero Terregino, Zeno Bisoffi, Elena Pomari

**Affiliations:** 1 Department of Infectious, Tropical Diseases and Microbiology, IRCCS Sacro Cuore Don Calabria Hospital, Negrar di Valpolicella, 37024 Verona, Italy; michela.deiana@sacrocuore.it (M.D.); chiara.piubelli@sacrocuore.it (C.P.); francesca.perandin@sacrocuore.it (F.P.); davide.treggiari@sacrocuore.it (D.T.); davide.martini@sacrocuore.it (D.M.); chesini.fabio@gmail.com (F.C.); andrea.angheben@sacrocuore.it (A.A.); zeno.bisoffi@sacrocuore.it (Z.B.); 2 Laboratory of Experimental Animal Models, Division of Comparative Biomedical Sciences, Istituto Zooprofilattico Sperimentale delle Venezie, 35020 Legnaro, Italy; fbonfante@izsvenezie.it (F.B.); cterregino@izsvenezie.it (C.T.); 3 Department of Diagnostics and Public Health, University of Verona, 37129 Verona, Italy

**Keywords:** SARS-CoV-2, coronavirus, subgenomic, saliva, vaccine, BNT162b2, transmission, real time RT-PCR, droplet digital PCR

## Abstract

SARS-CoV-2 infection was monitored in 1898 health care workers (HCWs) after receiving full vaccination with BNT162b2. Untill 30 June 2021, 10 HCWs tested positive for SARS-CoV-2 using real time RT-PCR, resulting in a 4-month cumulative incidence of 0.005%. The infection was mildly symptomatic in six (60%) and asymptomatic in four (40%) individuals. Among the infected HCWs, eight consenting individuals provided paired NPS and saliva during the course of infection, for the purpose of the analysis performed in the present study. Genomic and subgenomic viral RNAs were investigated using real-time RT-PCR in both biological specimens. The temporal profile of viral load was measured using ddPCR. Viral mutations were also analysed. Subgenomic viral RNA was detected in 8/8 (100%) NPS and in 6/8 (75%) saliva specimens at the baseline. The expression of subgenomic RNA was observed for up to 7 days in 3/8 (38%) symptomatic cases. Moreover, concordance was observed between NPS and saliva in the detection of viral mutations, and both N501Y and 69/70del (associated with the B.1.1.7 variant) were detected in the majority 6/8 (75%) of subjects, while the K417T mutation (associated with the P.1-type variants) was detected in 2/8 (25%) individuals. Overall, our findings report a low frequency of infected HCWs after full vaccination. It is, therefore, important to monitor the vaccinees in order to identify asymptomatic infected individuals. Saliva can be a surrogate for SARS-CoV-2 surveillance, particularly in social settings such as hospitals.

## 1. Introduction

The pandemic outbreak of SARS-CoV-2/COVID-19 infection led to a prompt effort to produce vaccines against the virus in a very short time period [1]. The European Commission had clearly indicated that health care workers (HCWs) must be privileged in the vaccine programme due to a significant risk of exposure [2]. In Italy, since the start of the vaccination campaign in December 2020 to 2 August 2021, 32,547,009 (60.26%) individuals over 12 have completed the vaccination cycle [3]. Vaccination reduces the risk of severe disease; however, little is known about virus carriage (including non-sterilising immunity). Thus, studies are required to provide evidence regarding the reduction in transmission by asymptomatic vaccinated individuals that are laboratory-confirmed as SARS-CoV-2-positive [4]. Indeed, the detection of SARS-CoV-2 has been reported in people who received one or both doses of the vaccine [5]. Thus, the rapid and accurate detection of SARS-CoV-2, as well as of viral infectivity, are essential in controlling the outbreak of infections [6]. Based on the literature, the detection of SARS-CoV-2 subgenomic RNA (sgRNA) in diagnostic samples can indicate active virus replication/transcription and recent infection [6,7,8,9,10,11]. Indeed, in the Coronaviridae family, including SARS-CoV-2, sgRNAs are replicative intermediates, and their abundance may reflect viral replication activity and the severity of host infection. After infecting the host cells, SARS-CoV-2 uses both replication and transcription to produce genomic RNA and sgRNA using a discontinuous transcription mechanism [12]. To date, limited studies have examined SARS-CoV-2 sgRNAs expression after full vaccination [13]. Thus, we aimed to investigate sgRNA expression during the course of SARS-CoV-2 infection in the vaccinated HCWs of our hospital. Moreover, based on the benefits of saliva testing as an alternative to nasopharyngeal swab (NPS) testing for diagnosing SARS-CoV-2 infection [14,15,16], we conducted the present study using both NPS and saliva specimens. Following local directives, since the start of the vaccination campaign at the beginning of 2021, infection surveillance was maintained for all the vaccinated HCWs through antigen testing or RT-PCR analysis on NPS, every week or every 21 days, depending on exposure risk. If they tested positive after full vaccination, all consenting HCWs were provided with additional NPS and paired saliva sampling for SARS-CoV-2 identification using RT-PCR. The aim of the present study was to closely monitor the incidence of SARS-CoV-2 infection, using genomic viral RNA detection, in subjects who were fully vaccinated. In order to infer the replication capacity of the virus in subjects that tested positive for SARS-CoV-2 after receiving the entire vaccination cycle, we investigated the sgRNA using RT-PCR. Viral mutations were also investigated. Moreover, NPS was compared with salivary testing for SARS-CoV-2 RNA detection.

## 2. Materials and Methods 

### 2.1. Setting of Study

The study was carried out at the IRCCS Sacro Cuore Don Calabria Hospital, Negrar, Verona, Italy, between 1 February 2021 and 31 June 2021. The study (No. 39528/2020 Prog. 2832CESC, 17 July 2020) was approved by the competent Ethics Committee for Clinical Research of Verona and Rovigo Provinces. Written informed consent was obtained from the patients and all research was performed in accordance with relevant guidelines/regulations. A total of 1898 fully vaccinated HCWs were periodically tested for SARS-CoV-2 infection using antigen tests (PANBIO™ COVID-19 Ag RAPID TEST DEVICE, Abbott, Chicago, IL, USA) or RT-PCR on NPS (Eswab, COPAN, Brescia, Italy). Individuals found to be positive for infection were subjected to an additional RT-PCR on NPS as a confirmatory test, and a saliva sample was taken (whole saliva was collected by drooling in a sterile plastic tube without preservative solution [17]). The collection was conducted at the following time points: 1 to 2 days after infection (T1), within 10 days post-infection (T2) and 10 to 15 days post-infection (T3).

### 2.2. RNA Extraction

RNA was isolated from 200 μL of NPS using an automated Microlab Nimbus workstation (Hamilton, Reno, NV, USA), coupled with a Kingfisher Presto system (Thermo Fisher Scientific, Waltham, MA, USA), using the MagnaMax Viral/Pathogen extraction kit (Thermo Fisher Scientific, Monza, Italy), according to the manufacturers’ instructions. An Automated Nextractor NX-48 with a Viral NA Extraction kit (Genolution Inc., Seoul, Korea) was used for saliva (200 μL). The isolated RNA was used for real-time RT-PCR and then stored at −80 °C for further analysis.

### 2.3. Reverse Transcriptase Real-Time PCR (RT-PCR)

**Figure 1:**
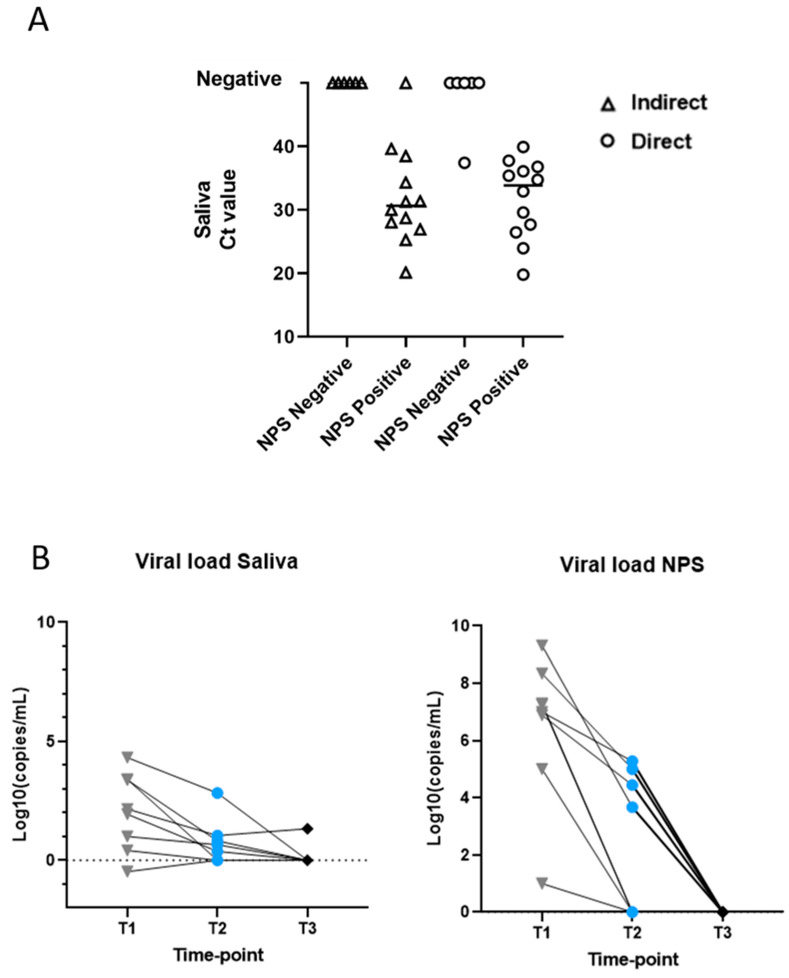
SARS-CoV-2 genomic RNA detection in saliva and NPS of 8 health care workers (HCWs) resulted positive after full vaccination. Dots represent data obtained from individual subjects (HCW n. 1–8) at the following time points: T1, 1–2 days after diagnosis; T2, 7–10 days after diagnosis; T3, 10–15 days after diagnosis. (**A**) Ct results of RT-PCR in saliva by indirect and direct approaches vs. NPS tested positive or negative for SARS-CoV-2 infection. For representation purposes, the undetected amplification of negative results in saliva is reported with Ct > 40. (**B**) Analysis of viral load variation during infection using ddPCR in NPS and saliva. Each line corresponds to individual HCWs’ viral load variation. Gray triangles correspond to the viral load at T1, blue dots to T2, and black rhombuses to T3. Data are represented as Log10copies/mL. For undetected viral load, value is reported equal to 0.

The routine diagnostics RT-PCR method was performed using the Bosphore Novel Coronavirus (2019-nCoV) Detection Kit v4 (Anatolia geneworks, Istanbul, Turkey), targeting N, Orf1ab and E target genes for NPS, as well as saliva specimens, in order to detect SARS-CoV-2 infection (Figure 1A). Regarding the viral sgRNA, the molecular test REALQUALITY SARS-CoV-2 SubG (AB ANALITICA, Padova, Italy) (targeting sgE/M and sgN) was used, following the manufacturer’s instructions. For the analysis of sgRNA expression, data were expressed as 2^−∆Ct^ (sgRNA Ct–gRNA Ct) in order to analyse sgRNA in relation to the genomic RNA (gRNA). In addition, the same data from sgRNA and gRNA were analysed individually in relation to human internal amplification control (RNaseP) and expressed as 2^∆∆Ct^ [18] [T2 or T3 (gRNA-RNaseP)–T1 (gRNA-RnaseP)] for the analysis of gRNA, and [T2 or T3 (sgRNA-RNaseP)–T1 (sgRNA-RnaseP)] for the analysis of sgRNA. Moreover, the RT-PCR method was used for the analysis of known viral mutations using the REALQUALITY SARS-CoV-2 Variants (AB ANALITICA, Padova, Italy) (targeting N501Y, K417T and K417N) and SARS-CoV-2 Nucleic Acid Mutation Diagnostic kit (Sansure Biotech Inc, Hunan, China) (targeting N501Y and HV69/70del), following the manufacturers’ instructions. All the RT-PCRs were performed on a CFX-96 Touch Real-Time PCR Detection System (Bio-Rad, Hercules, CA, USA) using white 96-well plates. All experiments were performed using a positive internal control of amplification, a positive control sample and a no-template control (NTC).

### 2.4. Direct Real-Time PCR

To achieve high sensitivity, saliva was also analysed using direct RT-PCR on crude saliva, using the bKIT Virus Finder COVID-19 (Hyris^Ltd^, London, UK) on a bCUBE^®^ (Hyris^Ltd^) portable instrument [19]. Crude saliva (50 μL) was mixed with the provided solution (the exact composition of the solution is not available as part of the intellectual property rights from Hyris^Ltd^) and incubated at 95 °C for 5 min for activation [20], and then immediately used for RT-PCR analysis, following the manufacturer’s instructions. The analysis was performed using a positive internal control of amplification, a positive control sample and a no-template control (NTC).

### 2.5. DdPCR-One Step Reverse Transcriptase

We performed the ddPCR analysis according to the manufacturer’s instructions from the 2019-nCoV CDC ddPCR triplex probe assay (dEXS28563542, Bio-Rad, Hercules, CA, USA), as previously described [21]. We used a negative control (NTC) and a positive control (a mixture of synthetic viral target N1&N2, and the human gene RPP30 as a control of amplification). The analyses were performed on a QX200 ddPCR system (Bio-Rad, Hercules, CA, USA). The reactions with less than 7000 droplets were repeated. Data were analyzed using the QX Manager 1.2 Standard Edition software (Bio-Rad, Hercules, CA, USA) and expressed as Log10 (copies/mL).

### 2.6. Graphical Representations 

Graphical representations were performed using GraphPad Prism v.8 software.

## 3. Results 

### 3.1. Setting of the Study

Since March 2020, all HCWs (2539 persons) were periodically tested for SARS-CoV-2 infection by RT-PCR on NPS. In January 2021, 1898 employees (82% of the employed subjects) were fully vaccinated with the BNT162b2 vaccine (Pfizer BioNTech, New York, NY, USA). A dramatic decrease in SARS-CoV-2 infection incidence in HCWs was observed after the dual-dose (21 days apart) vaccination program. Indeed, the percentage of new infections among HCWs decreased from 22% before vaccination to 0.2% 4 months after vaccination. In this context, 10 fully vaccinated HCWs (six males and four females; mean age 46 and 41 years old, respectively) were infected by the SARS-CoV-2 virus between 11 and 108 days after the second dose of the BNT162b2 vaccine. In the majority of the infected subjects (8/10), the source of infection was intra-familial due to contact with infected relatives. Data were unavailable for two individuals. None of these subjects had a previous SARS-CoV-2 infection and all produced IgG-RDB-S after vaccination, as determined by serology test (data not shown). None of the infected subjects were hospitalized and 6/10 (60%) reported mild symptoms. Among the 10 RT-PCR positive HCWs that were detected from the NPS sample, eight subjects also undertook a saliva test for SARS-CoV-2. Demographic and clinical data of these eight individuals are reported in the Supplementary File S1. The following results are based on this subset of HCWs.

### 3.2. Genomic Viral RNA Detection

Figure 1A reports the overall Ct results obtained from saliva during the course of infection (Ct ranged from 20.13 to 38.47). Data are compared to the total number of NPS that tested positive (Ct ranged from 14.88 to 34.27) or negative (amplification not detected) for SARS-CoV-2 infection. In particular, at baseline (T1), SARS-CoV-2 infection was detected in 7/8 (88%) saliva samples using indirect RT-PCR, whereas all specimens 8/8 (100%) resulted as positive through direct RT-PCR on crude saliva. Specifically, the specimen from individual n.7 at T2 resulted as positive using direct analysis, showing a Ct value of 39.91 for the N2 target gene. This was also confirmed by ddPCR detecting the N2 target gene signal in both saliva (1.4 Log10copies/mL) and NPS (5.28 Log10copies/mL). On the other hand, amplification was not detected in any of the saliva specimens that were paired with negative NPS, apart from a saliva sample (individual 8 at T2) that tested weak positive, with a Ct value of 37.43 for the N2 target gene, according to the direct method. The ddPCR method only detected a signal in saliva with 0.89 and 0.42 Log10copies/mL for N1 and N2 target genes, respectively. Moreover, ddPCR was used in order to monitor the viral load variation during the course of infection in all HCWs. It ranged from 9.32 to 1 Log10copies/mL in NPS, and from 4.32 to −0.48 Log10copies/mL in saliva, as reported in Figure 1B. All Ct and viral load values are reported in the Supplementary File S1.

### 3.3. Subgenomic Viral RNA Detection

**Figure 2:**
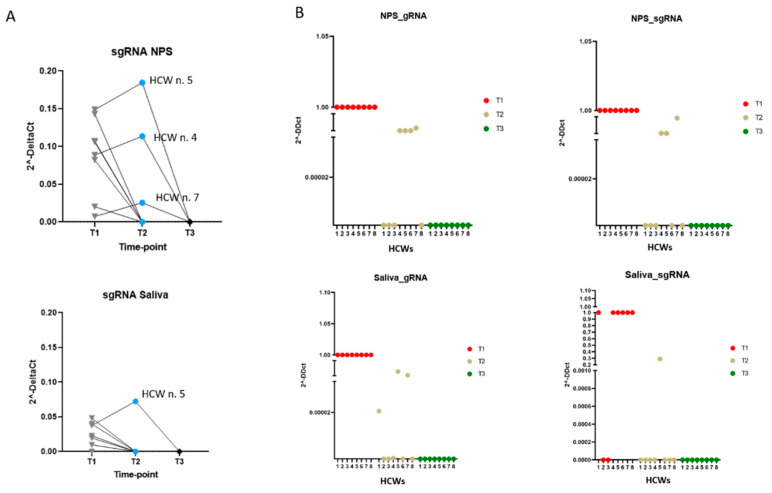
Subgenomic viral RNA analysis in saliva and NPS of 8 health care workers (HCWs) that resulted positive after full vaccination. Dots represent data obtained from individual subjects (HCW n. 1–8) at the following time points: T1, 1–2 days after diagnosis; T2, 7–10 days after diagnosis; T3, 10–15 days after diagnosis. (**A**) Subgenomic viral RNA (sgRNA) in relation to genomic viral RNA (gRNA). Data are expressed as 2^−∆Ct^ (sgRNA Ct–gRNA Ct). (**B**) Subgenomic (sg) and genomic (g) viral RNA analysed individually in relation to RNaseP (human internal amplification control). Data are expressed as 2^∆∆Ct^ (T2 or T3 (gRNA–RNaseP), T1 (gRNA–RnaseP) for the analysis of genomic RNA, and T2 or T3 (sgRNA–RNaseP), T1 (sgRNA–RnaseP) for the analysis of subgenomic RNA).

To investigate active viral replication, we performed RT-PCR to assess viral sgRNAs (sgE/M and N) in the clinical samples. All Ct values are reported in the Supplementary File S1. Considering the combined results for all sgRNAs analysed, in saliva, sgRNA was detectable in T1 in the majority of subjects (*n* = 6, 75%); whereas, in NPS, sgRNA was detected up to 10 days after diagnosis (T2). Specifically, in saliva, sgE/M ranged from 26.52 to 39.22 Ct in a total of five samples, and sgN ranged from 24.26 to 37.52 Ct in a total of six samples. Relating to NPS, sgE/M ranged from 25.82 to 40.18 Ct in a total of nine samples, and sgN ranged from 23.07 to 39.98 in a total of 11 samples. Moreover, the analysis did not reveal sgRNAs in all the specimens that tested negative for gRNA. For graphical representation, the total sgRNA (mean value of analysed sgRNAs) level of expression was compared to the viral gRNA in the same sample as 2^−∆Ct^ (sgRNA Ct–gRNA Ct) (Figure 2A). Overall, sgRNA was detected when gRNA ranged from 20.33 to 32.79 Ct in saliva, and from 21.12 to 35.01 Ct in NPS. In addition, sgRNA and gRNA were reported individually in relation to RNaseP (human internal amplification control) and data were expressed as 2^∆∆Ct^ [T2 or T3 (gRNA-RNaseP)–T1 (gRNA-RnaseP)] for the analysis of genomic RNA and [T2 or T3 (sgRNA-RNaseP)–T1 (sgRNA-RnaseP)] for the analysis of subgenomic RNA (Figure 2B). Individuals n. 4, 5 and 7 reported sgRNA in NPS, and only individual n. 5 reported sgRNA in saliva up to T2 (Figure 2A,B).

### 3.4. Viral Mutations Snalysis

Viral mutations (N501Y, 69/70del, K417T and K417N) were further investigated in both saliva and NPS using RT-PCR. The analysis was performed using two commercial kits (REALQUALITY SARS-CoV-2 Variants -AB ANALITICA, Padova, Italy- targeting N501Y, K417T and K417N and SARS-CoV-2 Nucleic Acid Mutation Diagnostic kit-Sansure Biotech Inc., Hunan, China- targeting N501Y and 69/70del) in order to combine the targeted mutations. The results between NPS and saliva were in agreement. The majority of individuals 6/8 (75%) were found to have both the N501Y and 69/70del mutations (associated with the B.1.1.7 variant), while 2/8 (25%) tested positive for the K417T mutation (associated with the the P.1-type variants). For the sake of clarity, the detection failed in two saliva specimens due to unavailable RNA data. Data are also reported in the Supplementary File S1.

## 4. Discussion 

Since the start of the vaccination campaign, the common goal was to guarantee widespread protection around the world; however, it is unlikely that the vaccine will be 100% effective at ending transmission or infection [4]. Thus, a potential risk is that fully vaccinated individuals can still get infected. Conversely, it is extremely important to manage and prevent new infections during the pandemic. In this study, we attempted to analyse the activity of SARS-CoV-2 transcription in infected HCWs after full vaccination with the BNT162b2 vaccine (Pfizer BioNTech). Through a combination of RT-PCR and ddPCR analyses, we investigated the gRNA and sgRNA expression in paired NPS and saliva specimens collected during the course of infection. In our cohort, along with a steep decrease in viral load in a short time period, we also observed a robust reduction in viral replication in the majority of cases. The subjects seemed to have active viral replication in their saliva when they were tested at the baseline, whereas the presence of replicating virus in NPS continued up to 10 days post-infection. In two HCWs with B.1.1.7 (one symptomatic with ageusia, anosmia, headache, and one asymptomatic) their saliva had a low gRNA and tested negative for sgRNA up to 2 days after diagnosis. However, their NPS tested positive for both sgRNA and gRNA. Conversely, one symptomatic (with nasal congestion) individual with B.1.1.7 showed the presence of SARS-Cov-2 virus for up to 7 days after infection in both saliva and NPS. Two others that were symptomatic (one with myalgia and the B.1.1.7 variant, and one with a fever 38.5 °C, myalgia and nasal congestion, and the K417T mutation, common in P.1-type variants) only showed a persistent viral sgRNA in NPS. The characterization of viral sgRNA in SARS-CoV-2 positive samples could be useful to better understand viral replication in the host cells. Whether persistent levels of sgRNA are actual proof of infectivity is still unclear; however, negative sgRNA can indicate that subjects are not infectious [22]. To date, previous studies reported evidence regarding the detection of viral sgRNAs using sequencing, RT-PCR and ddPCR, and all these findings were based on non-vaccinated cases [8,23,24]. The detection of viral sgRNA coupled with an increased viral load has been shown to be indicative of an aggravation of the disease [11]. Conversely, a reduction in viral load combined with a decrease in sgRNA might reflect a reduced risk of transmission to other susceptible contacts. Strafella and co-authors published a case report showing that the vaccine accelerated the resolution of viral infection and reduced transmission risk by decreasing the time of infectiousness [13]. However, this previous study was limited to a single HCW: a 38 years old man, fully vaccinated with BNT162b2, who tested positive in NPS at 54 days after vaccination, and for the N501Y and HV69-70del mutations associated with the B.1.1.7 lineage. He was completely asymptomatic and was monitored for 10 days after initial testing. Notably, the authors performed an evaluation of the temporal viral load decay of the HCW by comparing the temporal viral load decay of a reference group of 122 non-vaccinated subjects (including individuals with the wild-type and B.1.1.7 variant of SARS-CoV-2). The subject tested positive until 5 days after initial testing, showing a significant speed-up of viral decay in relation to the reference group. Relatedly, our findings showed a limited duration of viral shedding, with a decay in the NPS of symptomatic subjects for up to 7 or 10 days post-infection. On the other hand, our findings did not show a distinct impact of both viral genomic and subgenomic shedding dependently on viral mutations, although further analysis in a larger cohort is needed to better explore this potential occurrence.

Moreover, our data provide additional and confirmatory findings that saliva may be an alternative diagnostic sampling method to NPS for SARS-CoV-2 [25]. The direct RT-PCR showed higher sensitivity than the indirect method in revealing gRNA in all the saliva specimens, suggesting that saliva may be ideal for crude analysis, including for point-of-care testing. Relating to sgRNA detection, a higher speed of decay was observed in saliva compared to NPS, indicating the potential for virus-specific kinetics in the saliva [26].

In the context of COVID-19 vaccination campaigns, it is highly important to understand the base of evidence regarding vaccine effectiveness in preventing symptomatic and asymptomatic infections and transmission from infected individuals to susceptible contacts. Investigating both viral genomic and subgenomic RNAs in subjects infected with SARS-CoV-2 might be relevant for epidemiology purposes and infection control, with potential for clinical significance mainly related to the duration of the disease [24]. The detection of sgRNAs can provide useful data when combined with routine assays based on the detection of viral gRNA, particularly to assess the phase (early vs. late) of infection. The sgRNAs’ level of expression can be also used to clarify cases of dubious positivity (Ct > 35 of viral gRNA), principally in the late phase of infection, in order to better manage the containment measures. Overall, the present study suggests that protection can be achieved with the vaccination programme, combined with the appropriate mitigation measures, prompt testing, tracing and isolation. It further indicates the importance of ongoing vigilance regarding variants of concern (VOCs). As already observed in other studies [27], we found that the vaccination was effective in protecting HCWs from severe complications, although it was not perfect at preventing infection. This underlines the need to maintain the other measures of protection, particularly as variants persist, representing new challenges. Recent evidence shows that circulating IgG- and IgA- targeting the viral S protein declined over time, after the second dose of the SARS-CoV-2 mRNA-based vaccination [28,29]. Conversely, it has also been demonstrated that a prolonged germinal centre B cell response is induced, enabling a generation of robust humoral immunity [30,31]. Mild or no symptoms were reported in our cohort of vaccinees who were tested positive for SARS-CoV-2, suggesting that it provides protection against severe clinical complications. Additionally, short viral shedding, based on the viral genomic and subgenomic RNAs expression, can potentially indicate a mitigated viral replication with positive implications. This observation supports the data from the other literature reporting that vaccination reduced the chance of virus transmission [32].

However, this study has some limitations, such as the small sample size and that HCWs were only investigated up to 4 months after full vaccination. Therefore, sgRNA as intermediators of active viral replication should be be investigated in other large COVID-19 vaccinee populations in comparison with non-vaccinated populations. In addition, whether saliva samples can be used as a surrogate of protection in the long term after vaccination should also be further examined.

## 5. Conclusions

In conclusion, the low frequency of positive tests after full vaccination is auspicious; however, our data indicate the importance of maintaining public health mitigation measures, such as masking, physical distancing and regular testing, even with a high rate of vaccinees. It is, therefore, important to monitor the vaccinees in order to identify asymptomatic infected individuals. Saliva can be a surrogate for SARS-CoV-2 surveillance, particularly in social settings such as hospital and schools. Our results also emphasize the importance of tracking viral variants and more research is required to better understand the evolutionary impact on VOCs, and vaccine effectiveness against them, in order to prevent their spread.

## Data Availability

All data generated or analysed during this study are included in this published article (and its Supplementary Information Files).

## Supplementary Materials

The following are available online at https://www.mdpi.com/article/10.3390/microorganisms9081738/s1, File S1: demographic data of HCWs included in the present study and experimental data of RT-PCR and ddPCR analyses.
